# Successful difficult airway management of a child with Coffin‐siris syndrome

**DOI:** 10.1002/ccr3.1045

**Published:** 2017-06-29

**Authors:** Ahmet Selim Ozkan, Sedat Akbas, Mehmet Ridvan Yalin, Emine Ozdemir, Zeynep Koylu

**Affiliations:** ^1^ Department of Anesthesia University of Inonu Malatya Turkey

**Keywords:** Child, Coffin‐siris syndrome, difficult airway

## Abstract

Management of airway in patients who have Coffin‐Siris syndrome (CSS) is often problematic because most of these patients have difficult airway. NTI via C‐MAC VL is an useful alternative to direct laryngoscope for orotracheal intubation in airway and anesthetic management in a case of CSS. Alternative airway devices should be readily available.

## Introduction

Coffin‐Siris syndrome (CSS) is a rare genetic disorder that causes developmental and intellectual disability such as mental retardation, growth restriction, absent fifth‐digit fingernails or hypoplastic fifth finger terminal phalanx, coarse facial features, and cardiac/neurological/gastrointestinal/genitourinary anomalies 1. Because of micrognathia, macroglossia, hypotonia, and lax joint, airway management can be difficult in a child as well as mental problems due to poor communication 2. Especially, facial malformations may make intubation difficult. Management of airway in patients who have congenital syndromes such as CSS is often problematic because most of these patients have difficult airway.

After the first report 3, over 60 patient with CSS have been reported 4. The numbers of cases since then have increased and shown to be around 80. This is the first report that videolaryngoscope has been used in successful airway management of a child with CSS. We report a case of a 9‐year‐old child with CSS in whom airway management was difficult and problematic.

## Case Report

**Figure 1:**
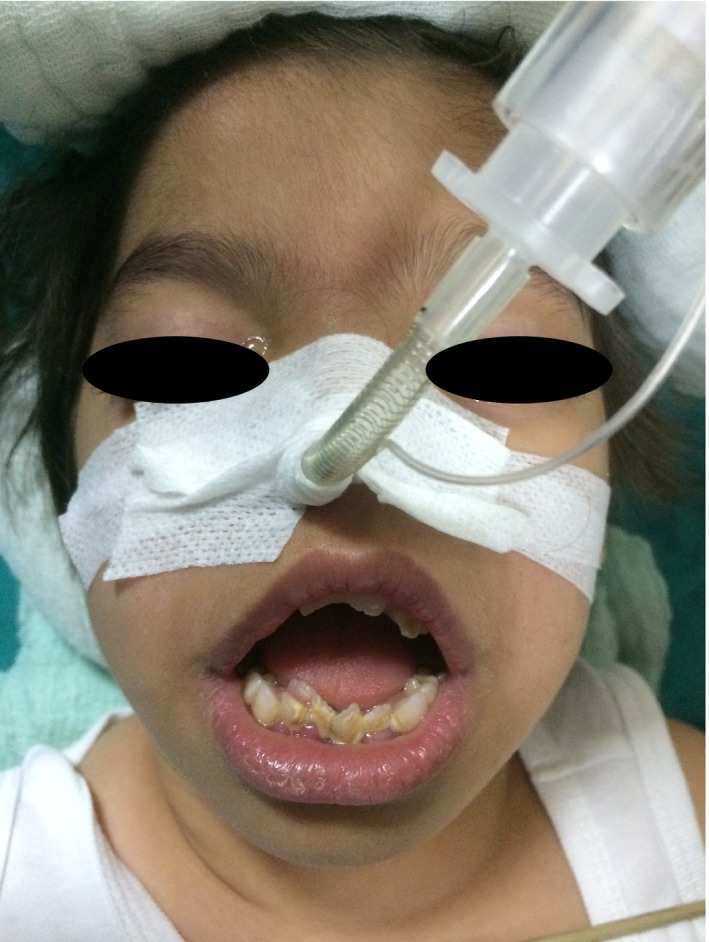
Coarse facies, flat nasal bridge, wide mouth, thick lips, macroglossia, micrognathia, and irregular and protruding teeth.

A nine‐year‐old child with CSS, a height of 90 cm and weighs 13 kg was scheduled to undergo dental restoration with general anesthesia. In preoperative assessment, mental retardation, cognitive growth disability, hypotonia, coarse facies reflected by flat nasal bridge, wide mouth, thick lips, macroglossia, micrognathia, and irregular and protruding teeth were recorded (Fig. 1). Airway evaluation was Mallampati class 3. Nasal endotracheal intubation (NTI) was planned for airway management. Midazolam 5 mg was given intranasal for premedication and patient admitted to the operating room. Continuous monitoring was performed by pulse oximetry, noninvasive blood pressure and electrocardiography. After oxygen breathing during 5 min, anesthesia was induced with 50 mg propofol intravenous (IV), 15 mcg fentanyl IV and after successful bag mask ventilation via facemask, rocuronium 8 mg IV was given. NTI was tried out at first under direct laryngoscopy with 3.5‐mm endotracheal spiral tube. Only epiglottis was occured and the cords were not visualized. Cormack‐Lehane score was 3. The endotracheal tube could not canalized to trachea using magil pens. Patient was ventilated easily between multiple intubation attempts and using of videolarygoscope (C‐Mac) was aimed for NTI. Same endotracheal tube was inserted to trachea with the aid of videolaryngoscope. During NTI, the cords were visualized successfully and patient was intubated without difficulty. Cormack‐Lehane score was 1 during videolaryngoscopy. NTI was confirmed by auscultation, chest movement and end‐tidal CO_2_ wave form. Three teeth extractions and 12 teeth fillings were done during general anesthesia. Operation and anesthesia were eventless. Paracetamol 150 mg IV was given for postoperative analgesia. Sugammadex 30 mg IV was given for neuromuscular block reversing at the end of surgery. After the spontaneous ventilation was returned, patient was extubated. After 1 day, she was discharged home.

## Discussion

Some of the typical features of CSS such as congenital anomalies causing airway difficulties can be of concern to anesthesiologists. Airway problems are important reasons due to anesthesia for mortality and serious morbidity. Conditions such as micrognathia and macroglossia may complicate airway management in these patients 5. Also, craniofacial and orofacial deformities can cause increased possible difficult airway. Difficult airway possibility increase with the age of the patient. Many genetic syndromes such as Down syndrome (atlantoaxial instability, macroglossia, subglottic stenosis) or Turner syndrome (micrognathia) can result in difficult airway because of more deformities. Laryngomalacia is the most common congenital laryngeal disease that causes inspiratuar stridor and airway obstruction 5. Collapse of trachea during recovery anesthesia may make intubation difficult cause to prolonged intubation and ventilation in this patient. The risk of aspiration increases in these patients because of mental retardation 6.

Coffin‐Siris syndrome is an autosomal recessive mode of inheritance and more common in females, but these patients are chromosomally normal 7. CSS is a rare syndrome and has reported in only a few patients since 1970. Macroglossia, choanal atresia, abnormal dentition, and short neck are observed in CSS and important for the management of the airway 8. Our patient has a syndrome called CSS. Coarse facies reflected by flat nasal bridge, wide mouth, thick lips, macroglossia, micrognathia, and irregular and protruding teeth were recorded in this case during preoperative assessment and Mallampati score was 3. So that necessary preparations were made such as alternative airway devices. Because of dental restruction, NTI was planned. Successful anesthesia management has been published in few of reported cases 8, but has not been reported NTI practice in airway management in these patients. No description about the airway management via NTI practice.

FOB was the most valuable device available for anesthesiologist to manage difficult airway, but recently VL has supplanted the FOB due to easily applicability. In these patients, FOB and VL could have been attempted to intubate the trachea successfully. Awake intubation via FOB or VL can be tried, but our patient was not available for this procedure due to be child. Altun et al. 9 informed that 5‐year‐old child was intubated via FOB and oral fiberoptic intubation has been done successfully in a patient with CSS who has difficult airway and laryngomalacia. But, VL is a good alternative airway device in difficult airway cases. NTI can be applied with VL successfully in the management of difficult airway.

In the most reported cases, we learned that corrective surgery such as cleft palate repair, cardiac procedures, hernia, adenoid removal etc. was applied in patients with CSS. This patient underwent a dental procedure. Dimaculangan et al. 10 noted that LMA was contemplated in airway management of CSS patient, undergoing dental restoration. After unsuccessful orotracheal and nasotracheal intubation, the case was canceled because of elective nature of the procedure and difficult airway. Another report was noted that LMA was inserted without difficulty in a chilf with CSS 11. In our patient, LMA was not used because of the nature of the procedure (dental surgery).

## Conclusion

We can conclude that NTI via C‐MAC VL is an useful alternative to direct laryngoscope for orotracheal intubation in airway and anesthetic management in a case of CSS. Airway problems in CSS patient can be of concern to anesthesiologist, but alternative airway devices and intubation methods can decrease this concern. Alternative airway devices should be readily available.

## Authorship

ASO and SA: involved in implementation of the process, collected data, wrote the manuscript. MRY, EO, ZK: Data collection, Article writing.

## Conflict of Interest

None declared.
